# Supplementary material for: Comparison of Oncologic Outcomes between Carbon Ion Radiotherapy and Stereotactic Body Radiotherapy for Early-Stage Non-Small Cell Lung Cancer
Source: Cancers (Basel). 2021 Jan 6;13(2):176. doi: 10.3390/cancers13020176 (PMC7825544; doi:10.3390/cancers13020176)
Supplement: Supplementary file 1 [file cancers-13-00176-s001.pdf]

cancers

MDPI

Supplementary Materials

Comparison of Oncologic Outcomes between Carbon Ion Radiotherapy and Stereotactic Body Radiotherapy for Early-Stage Non-Small Cell Lung Cancer

Yuhei Miyasaka, Shuichiro Komatsu, Takanori Abe, Nobuteru Kubo, Naoko Okano, Kei Shibuya, Katsuyuki Shirai, Hidemasa Kawamura, Jun-ichi Saitoh, Takeshi Ebara, Tatsuya Ohno

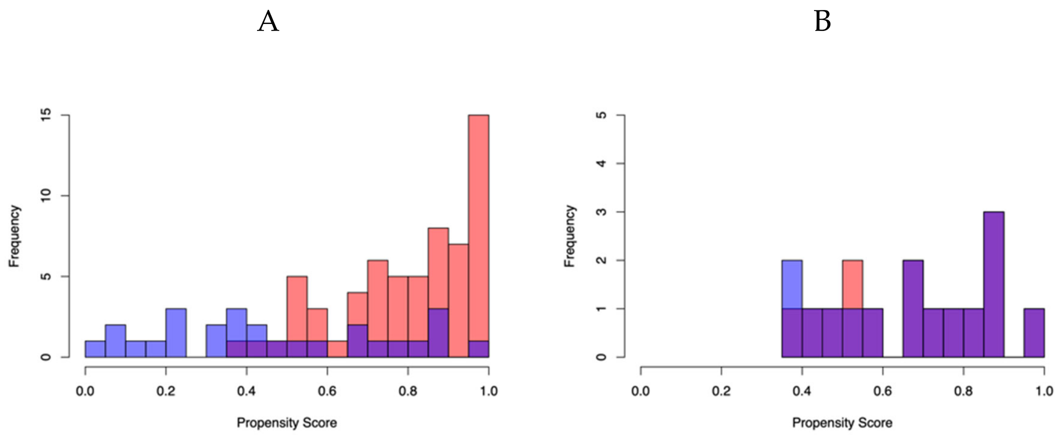

Figure S1. Distributions of the propensity scores. (**A**) The entire cohort and (**B**) the matched cohort. Red and blue columns show the carbon ion radiotherapy group and stereotactic body radiotherapy group, respectively.
